# Case Report: A False Negative Case of Anti-Yo Paraneoplastic Myelopathy

**DOI:** 10.3389/fneur.2021.728700

**Published:** 2021-10-22

**Authors:** Christopher M. Bartley, Neelroop N. Parikshak, Thomas T. Ngo, Jessa A. Alexander, Kelsey C. Zorn, Bonny D. Alvarenga, Min K. Kang, Massimo Pedriali, Samuel J. Pleasure, Michael R. Wilson

**Affiliations:** ^1^Weill Institute for Neurosciences, University of California, San Francisco, San Francisco, CA, United States; ^2^Department of Psychiatry and Behavioral Sciences, University of California, San Francisco, San Francisco, CA, United States; ^3^Department of Neurology, University of California, San Francisco, San Francisco, CA, United States; ^4^Department of Biochemistry and Biophysics, University of California, San Francisco, San Francisco, CA, United States; ^5^Operative Unit of Surgical Pathology, Azienda Ospedaliera-Universitaria, Ferrara, Italy

**Keywords:** paraneoplastic neurologic disease, myelopathy, anti-Yo antibodies, phage display, breast cancer, CDR2L

## Abstract

The development of autoimmune antibody panels has improved the diagnosis of paraneoplastic neurological disorders (PNDs) of the brain and spinal cord. Here, we present a case of a woman with a history of breast cancer who presented with a subacute sensory ataxia that progressed over 18 months. Her examination and diagnostic studies were consistent with a myelopathy. Metabolic, infectious, and autoimmune testing were non-diagnostic. However, she responded to empirical immunosuppression, prompting further workup for an autoimmune etiology. An unbiased autoantibody screen utilizing phage display immunoprecipitation sequencing (PhIP-Seq) identified antibodies to the anti-Yo antigens cerebellar degeneration related protein 2 like (CDR2L) and CDR2, which were subsequently validated by immunoblot and cell-based overexpression assays. Furthermore, CDR2L protein expression was restricted to HER2 expressing tumor cells in the patient's breast tissue. Recent evidence suggests that CDR2L is likely the primary antigen in anti-Yo paraneoplastic cerebellar degeneration, but anti-Yo myelopathy is poorly characterized. By immunostaining, we detected neuronal CDR2L protein expression in the murine and human spinal cord. This case demonstrates the diagnostic utility of unbiased assays in patients with suspected PNDs, supports prior observations that anti-Yo PND can be associated with isolated myelopathy, and implicates CDR2L as a potential antigen in the spinal cord.

## Introduction

The initial evaluation of subacute or chronic progressive myelopathy includes investigations into structural, metabolic, inflammatory, and vascular causes. Inflammatory etiologies include infections due to organisms such as *Treponema pallidum*, varicella zoster virus, HIV-1, and *Borrelia burgdorferi* as well as autoimmune diseases like multiple sclerosis, neuromyelitis optica spectrum disorders, and paraneoplastic neurological disorders (PNDs). The diagnosis of a PND is supported by the detection of a recent or new malignancy and can be confirmed by the identification of autoantibodies associated with a specific PND in the blood and/or cerebrospinal fluid (CSF) (1). Moreover, identification of a specific PND autoantibody can guide a targeted search for unidentified neoplasms.

As the number of diagnostic paraneoplastic autoantibodies has grown, panels have been developed that simultaneously test for multiple antibodies to facilitate clinical diagnosis and management. Testing is often performed in two stages, with initial screening by indirect immunofluorescence assay (IFA) and/or cell-based assays (CBAs) followed by confirmatory reflex testing (CBAs or immunoblotting) if the initial screen suggests the presence of a paraneoplastic antibody. Although PND panels are sensitive and specific, they test for a limited number of autoantibodies, and cases with a high degree of clinical concern for PND are often seronegative. To expand the ability to detect autoantibody targets in human disease, phage immunoprecipitation sequencing (PhIP-Seq) with a programmable peptide library representing the whole human peptidome was developed (2–5). PhIP-Seq has led to the identification of novel autoantibodies in undiagnosed PNDs, and high-resolution epitope mapping by deep mutational scanning with phage in previously characterized PNDs (2, 6, 7).

Here, we present a patient with sensory ataxia secondary to a myelopathy for whom clinical PND autoantibody screening was negative and therefore did not undergo reflex testing. Unexpectedly, research-based PhIP-Seq identified antibodies in the CSF against anti-Yo antigens cerebellar degeneration related protein 2 like (CDR2L) and cerebellar degeneration related protein 2 (CDR2) that were subsequently orthogonally validated.

## Case description

**Figure 1:**
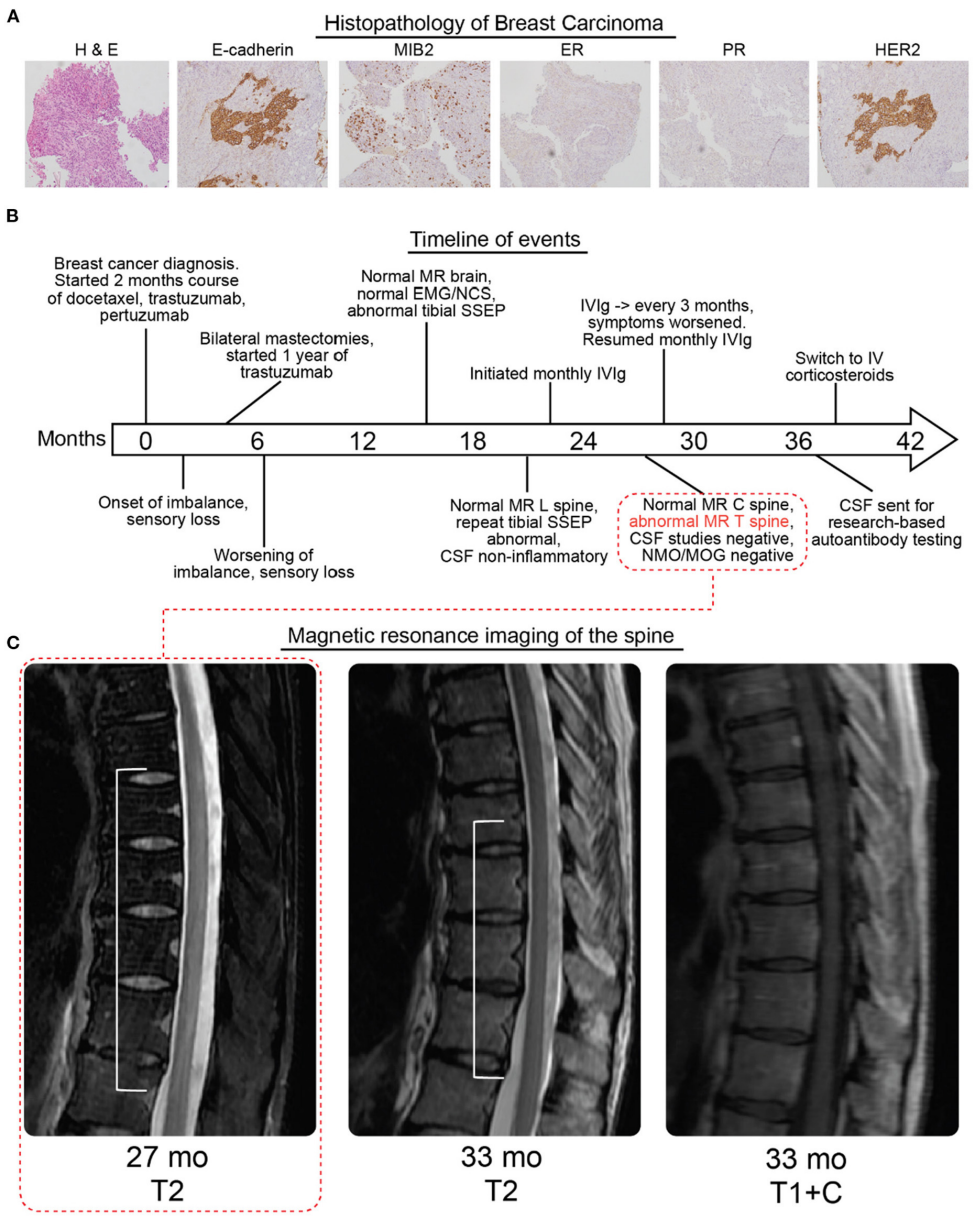
Histopathology, timeline, and radiologic findings. **(A)** Hematoxylin and eosin staining (left panel) followed by molecular characterization of patient breast carcinoma (MIB2, mindbomb E3 ubiquitin protein ligase 2; ER, estrogen receptor; PR, progesterone receptor; HER2, Erb-b2 receptor tyrosine kinase 2). The carcinoma was rated as E-cadherein and MIB2 expressing, ER/PR negative, and HER2 positive. **(B)** Timeline of diagnosis, symptoms, imaging, electrodiagnostic studies, and treatment. Imaging corresponding to 27 weeks is indicated in the first subpanel one of **(C)**. **(C)** MRI of the spine without gadolinium at 27 months showed a dorsally predominant T2 hyperintense signal spanning T5–T10. At 33 months there was some improvement with the T2 hyperintense signal now spanning T7–T10 which was not contrast enhancing (additional views and brain MRI in Supplementary Figure 1). All times are relative to the initial diagnosis of cancer, neurological symptoms began 2 months after the cancer diagnosis.

A 36-year-old woman with a history of breast cancer presented to our neurology clinic. She was previously diagnosed with infiltrating carcinoma of the left breast. Pathology had demonstrated an aggressive neoplasm with high nuclear grade and extensive perineoplastic lymphocytic infiltration [Figure 1A, WHO 2019 (8) classification “Invasive carcinoma of no special type,” T2N0, human epidermal growth factor receptor (HER2) positive, estrogen receptor, and progesterone receptor negative]. She was treated with pertuzumab, trastuzumab, and docetaxel for four cycles. Two months after her diagnosis and initiation of chemotherapy, she developed difficulty with her gait and sensory loss in her feet. Five months after her diagnosis, she underwent bilateral mastectomy, demonstrating no residual disease (Figure 1B). She subsequently completed a year of monthly trastuzumab.

Upon presentation to our clinic, 17 months after her diagnosis (15 months after the onset of her neurological symptoms), she noted difficulty maintaining balance when her eyes were closed and found it increasingly challenging to walk in a straight line. Her symptoms had progressed to the extent that she was now ambulating with the assistance of a cane. She endorsed mild sensory loss below her knees and denied any involvement of her upper extremities. She denied any vision changes, dysarthria, dysphagia, weakness, pain or bowel or bladder dysfunction.

Her general examination was unremarkable, and her neurological examination revealed no abnormalities with her mental status, cranial nerves, or strength. Sensory examination was notable for distal large fiber sensory loss, with reduced vibration below the knees with absent vibration in the feet and ankles and mild (10–20%) loss of light touch below her knees. Reflexes were present in the biceps and triceps but absent at her knees and ankles. The Romberg sign was present, and she had a wide-based, unsteady gait.

## Clinical course and diagnostic assessment

Evaluations prior to her presentation to our clinic were notable for normal contrast-enhanced magnetic resonance imaging (MRI) of her brain, and normal electromyography and nerve conduction studies (EMG/NCS). She had repeat EMG/NCS studies at our institution that were also unremarkable and unremarkable serological studies including a vitamin B12 level of 694 ng/L (Supplementary Table 1). She had normal median nerve somatosensory evoked potentials (SSEPs) from Erb's point, C5 spine and the cortex but abnormal tibial nerve SSEPs. With stimulation of the left posterior tibial nerve, a normal popliteal fossa response and P37 response could be seen (normal amplitude, latency, and inter-peak latency). However, the lumbar potential was poorly formed. With stimulation of the right posterior tibial nerve, a popliteal fossa, lumbar potential, and P37 response could be seen, but the latency to peak of the P37 component as well as the inter-peak latency between the popliteal fossa, lumbar potential and the P37 were delayed.

Given her exam and electrodiagnostic study results, a localization to the lumbosacral sensory nerve roots was initially favored. To further investigate, she had a lumbar puncture and a contrast enhanced MRI of the lumbar spine. CSF studies were without evidence of inflammation or malignant cells (Supplementary Table 1), and imaging did not identify any abnormalities in the lumbar spine or nerve root enhancement as is typically seen in chronic immune sensory polyradiculopathy (CISP). Her chemotherapy exposure was also considered as a potential cause. However, pertuzumab does not have any established neurotoxic effects. Trastuzumab and docetaxel are known to cause a polyneuropathy, but there was no evidence for a polyneuropathy on exam or EMG/NCS (9).

Three months after her initial visit, she reported worsening symptoms. On exam, her loss of sensation to light touch had worsened below her knees (50–60% loss) and now extended to mild involvement of her thighs. Vibration was absent up to her hip, and her gait instability was more prominent. Given her worsening symptoms and history of malignancy, inflammatory conditions affecting the spinal cord and/or nerve roots were still part of the differential diagnosis despite a negative work-up thus far. She was started empirically on monthly intravenous immunoglobulin (IVIg, total dose of 2 g/kg divided over 3 days). After three doses of IVIg, she reported stabilization in her sensory loss and felt her gait had improved. On monthly IVIg, she reported improvement in sensation above her knees. When treatment frequency was reduced to every 3 months, her symptoms worsened, prompting return to the monthly IVIg (Figure 1B).

A serum autoimmune encephalopathy panel was sent prior to IVIg administration, including for PNDs associated with sensory neuropathies and neuronopathies (such as anti-CRMP5 and anti-ANNA1), and was unremarkable (Mayo Clinic, Rochester, MN, test ID: ENS2) (10). An antibody panel for autoimmune etiologies of neuromuscular syndromes was sent after two doses of IVIg and revealed low titers of IgG antibodies to beta-tubulin, high titers of IgM antibodies to tri-sulfated heparin disaccharide (TS-HDS), and low titers of IgG antibodies to fibroblast growth factor receptor 3 (FGFR3) (Washington University, St. Louis, MO) (Supplementary Table 1). This pattern of abnormalities has been associated with sensory axonal neuropathies, but it is not specific (11, 12).

Repeat imaging of the brain, and first-time MRI of the cervical and thoracic spine without gadolinium were performed 27 months after her cancer diagnosis (25 months after onset of neurological symptoms), revealing a longitudinally extensive, dorsally predominant T2 hyperintensity from T5 to T10 (Figure 1C, Supplementary Figure 1). Repeat imaging of the thoracic spine with contrast 33 months after her cancer diagnosis and after receiving IVIg for about 1 year demonstrated less conspicuous and less extensive T2 signal from T7 to T10 with no contrast enhancement.

Two additional CSF exams showed no pleocytosis, no oligoclonal bands, no malignant cells, and a normal IgG index. Testing for anti-AQP4 and anti-MOG antibodies in the serum was negative (Supplementary Table 1). CSF was sent for an autoimmune encephalopathy panel and was negative (Mayo Clinic, test ID ENC2).

## Identification of candidate anti-Yo antibodies by phage immunoprecipitation sequencing

**Figure 2:**
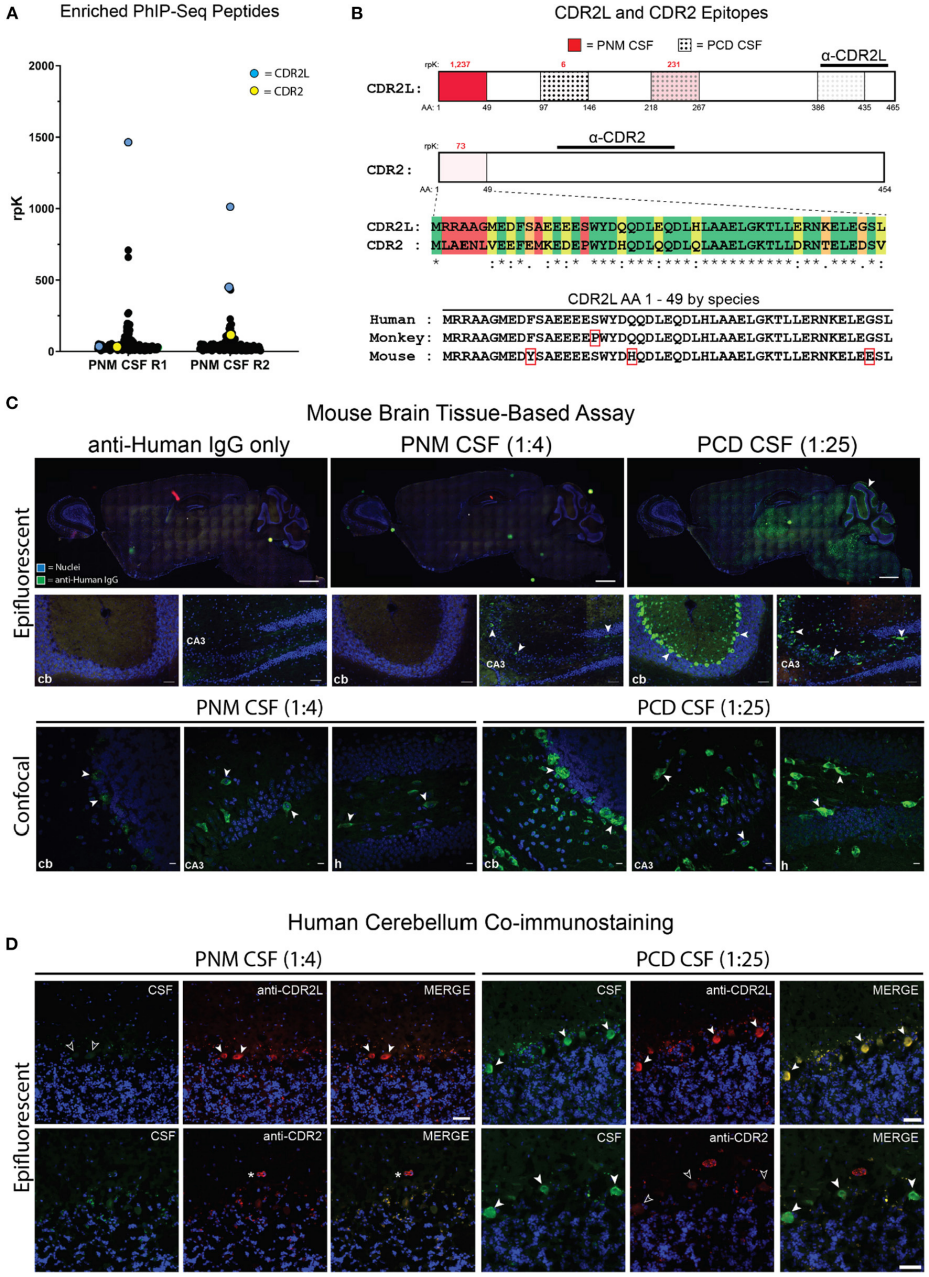
Identification of anti-CDR2L and anti-CDR2 antibodies and PNM CSF tissue-based assay. **(A)** Dot plot of individual PhIP-seq-identified peptides that were enriched in both technical replicates at least 10-fold above reference samples (R1 and R2, replicates 1 and 2, respectively). The most enriched peptide in each replicate mapped to CDR2L (blue points). A CDR2 peptide (yellow) was also identified. PhIP-Seq data for all other enriched peptides (black) are available in Supplementary Table 2. **(B)** CDR2L and CDR2 proteins and relevant epitopes. The intensity of red shading is proportional to the enrichment of that peptide by PNM CSF and the rpK are in red above each of the 3 detected CDR2L peptides and the single enriched CDR2 peptide. The dotted regions (3 rightmost) indicate the peptides that were enriched by PCD CSF whereby density of the dots represents the degree of enrichment. The immunogens for CDR2L and CDR2 commercial antibodies used for immunostaining are indicated by horizontal lines. A sequence alignment for the first 49 amino acids for CDR2L and CDR2 is shown [green, identical amino acids (AA); yellow, chemically similar AA; orange, minimally similar AA; and red, dissimilar AA]. The sequence alignment for the first 49 amino acids of human, monkey, and murine CDR2L are shown. Red rectangles indicate AA that differ from human. **(C)** Epifluorescent and confocal imaging of murine brain tissue immunostained with PNM CSF at 1:4 or PCD CSF at 1:25. cb, cerebellum; CA3, cornu Ammonis 3 of the hippocampus. Scale bars = 1,000 μm for whole brain sagittal images, 50 μm for epifluorescent hippocampal and cerebellar images, and 10 μm for confocal images. **(D)** Epifluorescent images immunostaining of human cerebellum by PNM and PCD CSF at 1:4 and 1:25, respectively. Arrows indicate Purkinje cells (PCs). In the upper left set of images, PNM CSF failed to immunostain CDR2L immunostained PCs (unfilled arrows). On the lower right, anti-CDR2 failed to immunostain PCs immunostained by PCD CSF (unfilled arrows). The asterisk indicates a blood vessel immunostained by anti-CDR2. Scale bars = 50 μm.

Given the patient's apparent response to IVIg, history of breast cancer, and negative clinical autoantibody testing, we screened her CSF for novel candidate autoantibodies using PhIP-Seq. Our phage display library is comprised of 49 amino acid peptides with a 25 amino acid overlap that together encode all known and predicted human proteins and their isoforms (7). To pan for candidate autoantibodies by PhIP-Seq, CSF is incubated with the PhIP-Seq library, IgG binds to target peptides displayed by individual T7 bacteriophage, IgG is isolated using protein A/G magnetic beads, and IgG-bound phage are sequenced to determine which human peptide they encoded. Unexpectedly, the N-terminal peptide for CDR2L, the major antigen in anti-Yo paraneopalstic syndrome, was the most enriched peptide by PhIP-Seq in both technical replicates (Figure 2A) (13). Two additional CDR2L peptides were also detected but less enriched, as was the N-terminal peptide of CDR2 (Figure 2B). Peptides to other known paraneoplastic autoantigens were not enriched (Figure 2A and Supplementary Table 2).

## Screening for anti-Yo antibodies by tissue-based assay

Routine clinical testing for anti-Yo antibodies is often performed by indirect immunofluorescent immunostaining of non-human primate cerebellar tissue. If the immunofluorescence assay (IFA) is positive, reflex immunoblotting of recombinant CDR2 is performed. Because CDR2L and CDR2 peptides were enriched by PhIP-Seq, we screened this patient's CSF for Purkinje cell immunoreactivity by sagittal mouse brain tissue-based assay. As a positive control, we used CSF from a previously reported case of clinically diagnosed anti-Yo paraneoplastic cerebellar degeneration (PCD CSF) that enriched multiple CDR2L peptides by PhIP-Seq [patient 03, (7)] (Figure 2B). At a 1:25 dilution, control PCD CSF robustly immunostained cerebellar Purkinje cells as well as sparse cerebral and hippocampal neurons (Figure 2C and Supplementary Figure 2).

In contrast, CSF from our patient with paraneoplastic myelopathy (PNM CSF) did not immunostain Purkinje cells when performed in technical triplicate and assessed by epifluorescence microscopy at a 1:4 dilution, consistent with the negative clinical testing (Figure 2C). Likewise, PNM CSF did not immunostain murine Purkinje cells even after antigen retrieval (Figure 2C). However, in one replicate PNM CSF did immunostain hippocampal neurons in a pattern similar to PCD CSF, albeit more sparsely (Figure 2C). We further characterized immunopositive replicates by confocal microscopy and found that immunostained hippocampal neurons were morphologically similar to those immunostained by PCD CSF, and that previously undetectable Purkinje cells were also faintly immunostained (Figure 2C).

We next performed coimmunostaining with antibodies that have been validated as specific to CDR2L and CDR2. In the cerebellum, the control PCD CSF colocalized with CDR2L and CDR2 staining in Purkinje cells. However, only anti-CDR2L staining co-localized with the control PCD CSF immunostaining of neurons in the molecular layer of the cerebellum. Likewise, PCD CSF and the anti-CDR2L antibody colocalized with hippocampal immunostaining. In contrast, hippocampal CDR2 expression was limited to arteriolar smooth muscle cells, consistent with CDR2 expression as annotated in the Human Protein Atlas (Supplementary Figure 2; http://www.proteinatlas.org) (14).

Mouse and human CDR2L are 93% similar by amino acid sequence, however some of the sequence differences are within the peptide that was most enriched by PNM CSF by PhIP-Seq (Figure 2B). To ensure the absence of immunostaining was not due to species differences, we co-immunostained human cerebellar tissue with PNM CSF or PCD CSF and anti-CDR2L or anti-CDR2 antibodies. As with mouse, PNM CSF at a 1:4 dilution did not appreciably immunostain human Purkinje cells while PCD CSF was strongly reactive at a 1:25 dilution and colocalized with CDR2L but not CDR2 (Figure 2D).

Taken together, these data suggest that some anti-Yo antibodies are not readily detected by Purkinje cell immunostaining—the standard initial screen for anti-Yo antibodies. Moreover, extracerebellar immunostaining may indicate the presence of anti-Yo antibodies, even in the absence of Purkinje cell immunoreactivity.

## Validation of Anti-Yo Antibodies in patient CSF

**Figure 3:**
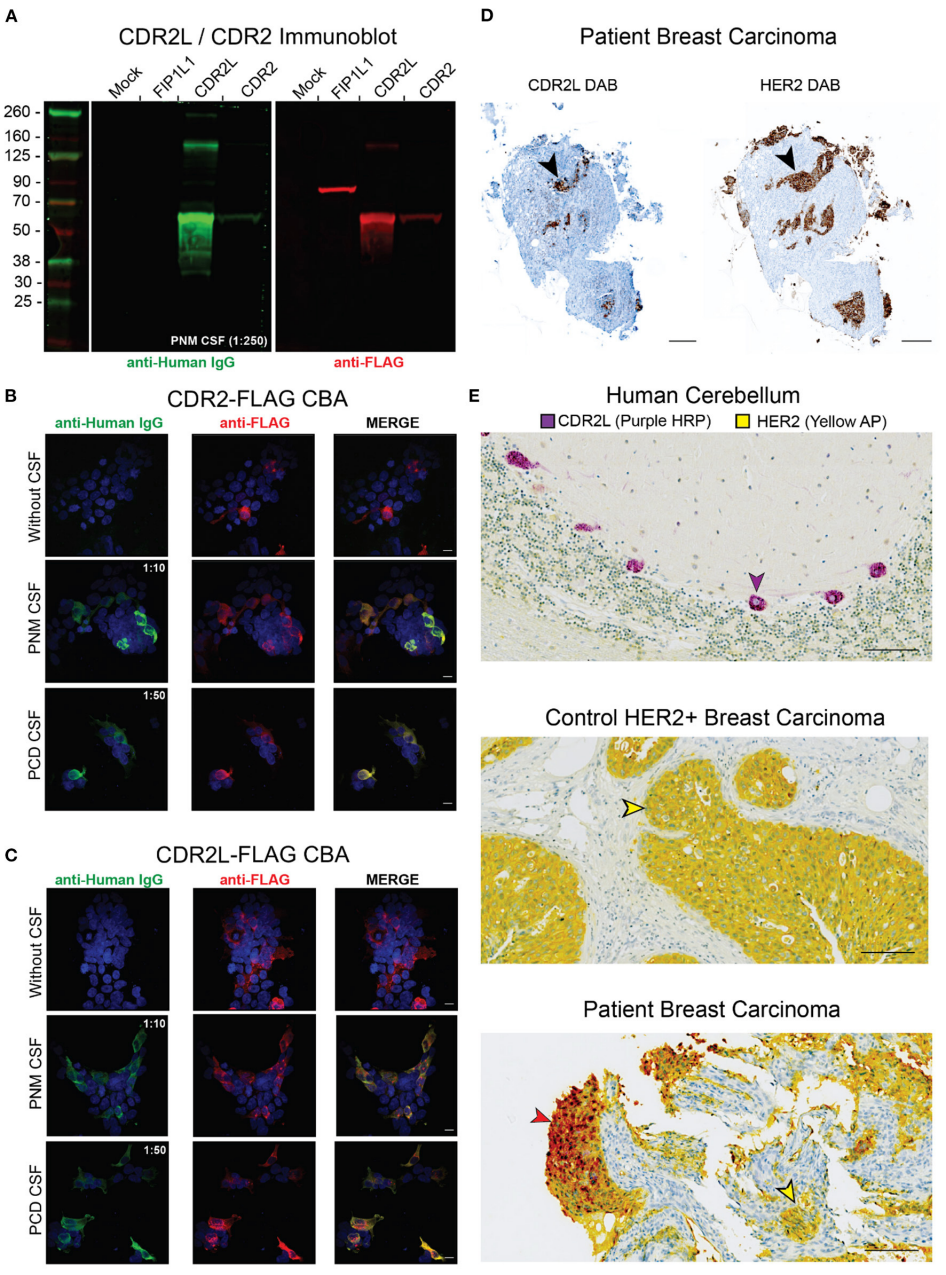
Validation of anti-Yo antibodies in PNM CSF. **(A)** HEK293T cells were transfected with FIP1L1-FLAG, CDR2L-FLAG, or CDR2-FLAG. Lysates were separated by SDS-PAGE and resulting immunoblots were probed with PNM CSF (green, 1:250) and counterstained with an anti-Human IgG 800CW secondary antibody (green). The membrane was the probed with anti-FLAG and anti-rabbit 680RD secondary antibody (red). PNM CSF IgG recognized recombinant CDR2L and CDR2 but not FIP1L1. **(B)** HEK293T cells were transfected with CDR2-FLAG and immunostained with PNM CSF at 1:10, PCD CSF at 1:50 (both green) and an anti-FLAG antibody (red). Scale bars are 10 μm. **(C)** HEK293T cells were transfected with CDR2L-FLAG and immunostained with PNM CSF at 1:10, PCD CSF at 1:50 (both green) and an anti-FLAG antibody (red). Scale bars are 10 μm. **(D)** 3,3′-Diaminobenzidine (DAB) immunohistochemistry of serial sections of the patient's breast carcinoma tissue for CDR2L (left) and HER2 (right). Black arrowheads highlight a corresponding area of DAB staining on serial sections. Scale bars = 200 μm. **(E)** Dual chromophore immunohistochemistry for CDR2L (purple HRP, horse radish peroxidase) and HER (yellow AP, alkaline phosphatase) on control cerebellum (top), control HER2 breast tissue (middle), and the patient's breast carcinoma (bottom). The purple arrowhead indicates a CDR2L+/HER2– Purkinje cell in the top panel. The yellow arrowheads indicate CDR2L–/HER2+ cells in control breast carcinoma (middle) and the patient's breast carcinoma (bottom). The brick red arrow indicates CDR2L+/HER2+ cells in the patient's breast carcinoma (bottom). Red results from the co-localization of the yellow and purple chromogens. Scale bars = 100 μm.

Because PNM CSF was not immunoreactive to Purkinje cells, we tested for anti-Yo antibodies by western blot. HEK 293T cells were either untransfected or transfected with C-terminal flag-tagged CDR2L (CDR2L-FLAG), CDR2 (CDR2-FLAG), or FIP1L1 (FIP1L-FLAG) as a non-target negative control. HEK 293T cell lysates were separated by SDS-PAGE and probed with PNM CSF at a 1:250 dilution and a commercial anti-FLAG antibody. PNM CSF IgG bound CDR2L and CDR2, but not untransfected HEK293T cell lysate or FIL1LP1-FLAG (end point dilution of 1:1,000 for PNM CSF not shown) (Figure 3A).

To further validate anti-CDR2L and anti-CDR2 autoantibodies, we tested PNM CSF for anti-Yo antibodies by HEK 293T overexpression CBA. HEK 293T cells were mock transfected, transfected with CDR2L-FLAG, or transfected with CDR2-FLAG, and immunostained with either PNM CSF (1:10) or PCD CSF as a positive control (1:50). Consistent with our immunoblot results, both PNM and PCD CSF immunostained CDR2L and CDR2-overexpressing HEK 293T CBAs (end point dilution of 1:100 for PNM CSF not shown) (Figures 3B,C).

Therefore, despite nearly undetectable immunostaining of Purkinje cells, western blot and CBA demonstrated the presence of anti-CDR2L and anti-CDR2 autoantibodies in PNM CSF.

## CDR2L expression is restricted to HER2+ cells in patient breast carcinoma tissue

HER2 is overexpressed in up to 96% of breast cancers from anti-Yo PCD patients compared to 15–20% of all breast cancers (15, 16). Additionally, CDR2L is expressed in up to 100% of ovarian carcinomas from patients with anti-Yo PCD and is overexpressed relative to control ovarian carcinoma tissue (17). Consistent with these molecular associations, we found that CDR2L and HER2 were expressed in the same anatomic regions when comparing serial sections of the patient's breast carcinoma tissue (Figure 3D). To determine whether CDR2L expression in our patient's tissue was restricted to HER2-expressing cells, we optimized CDR2L and HER2 for dual chromophore immunohistochemistry on control human cerebellum and control HER2+ breast carcinoma tissue from a patient not known to have anti-Yo PCD. We found that CDR2L expression in our patient's breast carcinoma colocalized with, and was restricted to, HER2+ carcinoma cells (Figure 3E).

## CDR2L and CDR2 protein expression in murine and human spinal cord

**Figure 4:**
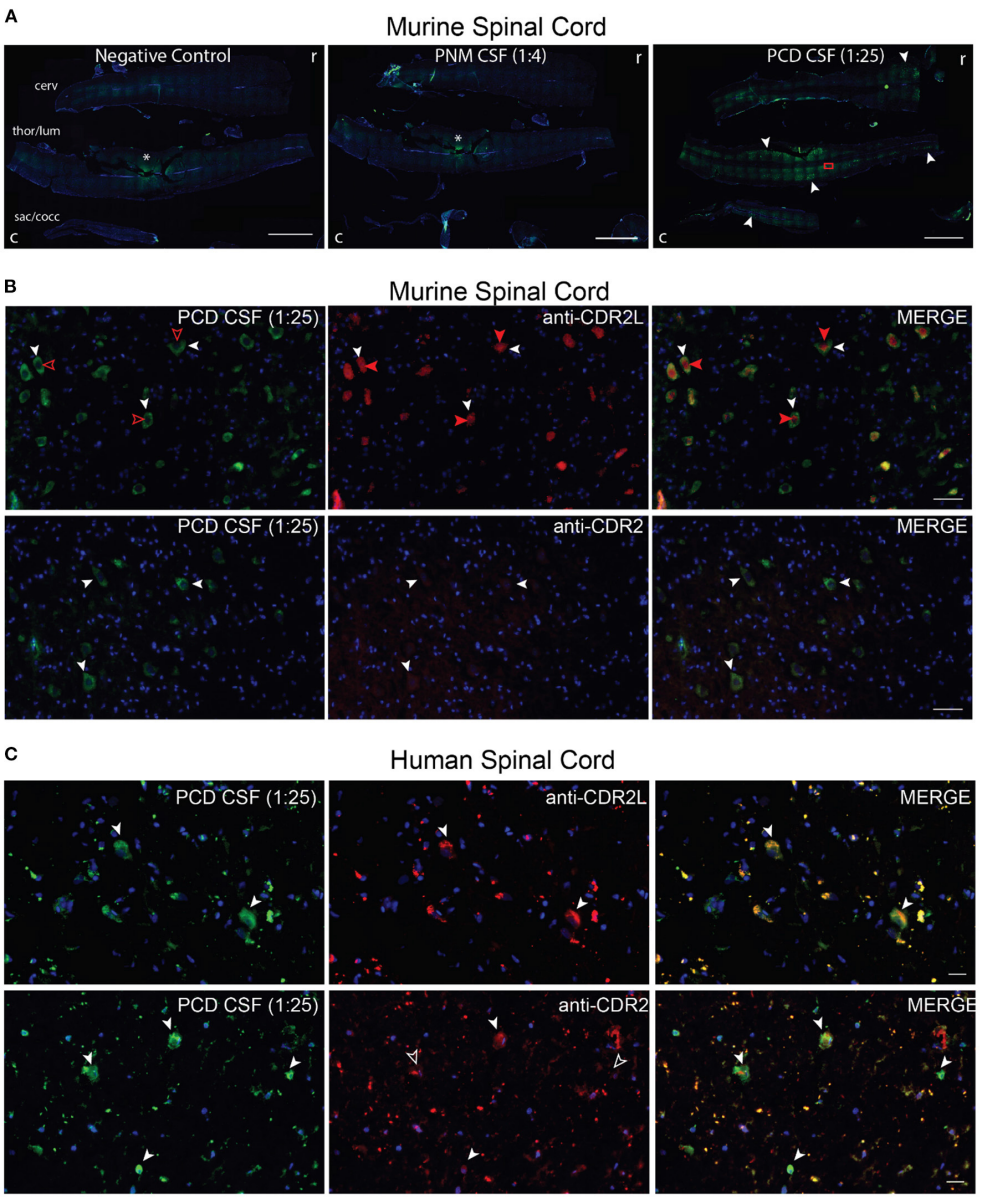
PNM CSF, PCDF CSF, CDR2L and CDR2 immunostaining of spinal cord. **(A)** Cervical (cerv), thoracolumbar (thor/lum), sacrococcygeal (sac/cocc) murine spinal cord was immunostained with PNM CSF (1:4) or PCD CSF (1:25). The rostral (r) and caudal (c) ends are on the right and left of each image, respectively. PNM CSF did not immunostain murine spinal tissue above background (asterisk = tissue artifact also seen in the negative control). PCD CSF immunostained cells along the longitudinal axis of the spinal cord (filled arrows). The red rectangle indicates the approximate region of images shown in **(B)**. Scale bars = 2,000 μm. **(B)** Coimmunostaining of murine spinal cord with PCD CSF (1:25) and anti-CDR2L or anti-CDR2. PCD CSF and anti-CDR2L immunostained the cytoplasm of large cells and colocalized with cytoplasmic anti=CDR2L immunostaining (white arrowheads). However, anti-CDR2L also immunostained nuclei (filled red arrows) but PCD CSF did not (unfilled red arrowheads). Scale bars = 200 μm. **(C)** Coimmunostaining of human spinal cord with PCD CSF. PNM CSF failed to immunostain human spinal cord (not shown). PCD CSF immunostained sparse cells that were also CDR2L positive (top row, white arrowheads). In the lower row, PCD CSF cells are costained by anti-CDR2L (white arrowheads). In the lower row, only some cells immunostained by PCD CSF were also immunostained by anti-CDR2 (filled arrow heads, co-stained cells; unfilled, PCD CSF exclusive cells). Scale bars = 20 μm.

Given that our patient presented with a myelopathy, we next asked whether PNM CSF anti-Yo antibodies bind to spinal cord tissue. We first immunostained mouse spinal cord sections with PCD CSF, PNM CSF, and anti-CDR2L and anti-CDR2 antibodies. As with cerebellar tissue, PNM CSF did not immunostain murine spinal cord above background fluorescence. In contrast, PCD CSF immunostained large cells along the longitudinal axis of the murine spinal cord (Figure 4A).

According to the Human Protein Atlas, CDR2L gene expression is higher than CDR2 in the human spinal cord; however, to our knowledge, CDR2L and CDR2 proteins have not been characterized in the murine or human spinal cord. In the murine spinal cord, PCD CSF immunostaining was restricted to the cytoplasm of large cells. In contrast, CDR2L immunostaining was both cytoplasmic and nuclear; however, all PCD CSF-immunostained cells were co-stained with CDR2L and vice versa (Figure 4B). In contrast, the anti-CDR2 antibody was poorly immunoreactive to neural cells but again strongly stained arteriolar smooth muscle cells (Figure 4B). In the human spinal cord, PCD CSF immunostaining also colocalized with anti-CDR2L more so than with anti-CDR2 immunostaining (Figure 4C). As with other tissue-based assays, PNM CSF failed to immunostain human spinal cord (data not shown).

## Follow-Up and Outcomes

Symptomatically and radiologically, the patient responded to IVIg treatment initially, but she had worsening symptoms 17 months later. Given the newly found thoracic myelopathy, which was suspected to be inflammatory in origin, she was switched to monthly pulsed dose steroids with subsequent stabilization. Small studies evaluating plasma exchange, IVIg, high dose corticosteroids, cyclophosphamide, and rituximab have been conducted in anti-Yo PCD, and no single agent has shown consistent efficacy (18). Furthermore, a case series of isolated paraneoplastic myelopathy associated with autoantibodies other than anti-Yo responded poorly to immunotherapy resulting in significant disability (19). Therefore, her mild improvement with immunotherapy was atypical for isolated paraneoplastic myelopathy. Her improvement also coincided with the remission of her breast cancer. Given that our patient had completed anti-tumor therapy and demonstrated a potential response to IVIg and corticosteroids, we transitioned her to rituximab as a steroid-sparing agent.

## Discussion

Here we presented a woman with a history of breast cancer and antibodies against anti-Yo antigens CDR2L and CDR2 despite negative clinical testing. Nearly 40% of PCD cases test negative for previously classified autoantibodies, and seronegative paraneoplastic myeloneuropathy has been reported in breast cancer (20). Serum and CSF that screen positive for anti-Yo antibodies by tissue-based immunostaining but only have anti-CDR2L antibodies (not anti-CDR2) are at risk for a false negative result because only CDR2 is included in confirmatory reflex assays (13, 21, 22). In this instance, initial screening of our patient's CSF for PND autoantibodies by IFA was negative, so presumably no confirmatory reflex assays looking for antibodies to either CDR2 or CDR2L were performed. Moreover, in contrast to many clinically diagnosed anti-Yo syndromes, our patient's CSF lacked oligoclonal bands and had a normal cell count, protein level, and IgG index (23). By maintaining a high suspicion for an inflammatory condition and applying unbiased high-resolution whole human proteome PhIP-Seq, we identified CDR2L and CDR2 antibodies in the CSF that we subsequently confirmed by immunoblot and CBA.

These data suggest that anti-Yo antibody detection depends on whether and how the target epitopes are represented in the diagnostic assays. In our patient's case, PhIP-Seq, immunoblot, and CBA were more sensitive than rodent brain immunohistochemistry. These findings may suggest that some CDR2 and CDR2L epitopes are inaccessible *in situ* because they are buried within a folded protein or shielded by protein-protein interactions. Indeed, other research studies have identified anti-Yo PND cases whose CSF screened negative for CDR2L/CDR2 antibodies by IFA (24, 25). Although we were able to detect anti-Yo antibodies in our patient's CSF at a 1:100 and 1:1,000 dilution by overexpression CBA and immunoblot, respectively, we cannot rule out that the antibody titer was below the limit of detection for tissue-based IFA (<1:240 for serum or <1:2 for CSF based on clinical testing parameters) where the effective concentration of CDR2L and CDR2 may be lower. In either case, our patient highlights the potential for false negatives when using tissue staining as a threshold assay.

Most cases of PNM are associated with antibodies against amphiphysin, Hu, or CV2/CRMP5, while anti-Yo antibodies are typically associated with PCD (19). Anti-Yo PCD with superimposed myelopathy is uncommon (24, 26–28) and isolated anti-Yo myelopathy [(24), case 7] or myeloneuropathy rarer still (10). It is unknown why some anti-Yo patients lack obvious cerebellar findings on exam though this does not preclude subclinical cerebellar dysfunction. This is certainly possible in the patient reported here though the structural imaging of the cerebellum was normal, and it was felt that the extensive dorsal column lesion in the thoracic cord accounted for her neurologic deficits.

In anti-Yo PCD, malignant expression of CDR2L and CDR2 elicits an immunologic response as evidenced by B and T cell inflammatory tumor infiltrates and CDR2L protein deposits in tumor-associated tertiary lymphoid structures (17). Endogenous Purkinje cell expression of CDR2L and CDR2 provides a direct pathogenic link, as inflammatory lymphocytes have been documented in post-mortem cerebellar tissue from patients with anti-Yo PCD (29). Demyelination and vacuolization of spinal cord tissue has been reported in a patient with anti-Yo PCD (29), but to our knowledge CDR2L and CDR2 protein expression in the human spinal cord and the myelopathology of anti-Yo PNM have not been further characterized. We detected CDR2L protein expression in the murine and human spinal cord that colocalized with anti-Yo antibodies from a patient with PCD. In contrast, CDR2 expression was primarily limited to arterioles and was poorly colocalized with anti-Yo antibodies. This finding is concordant with the growing literature suggesting that CDR2L is likely the pathogenic antigen in anti-Yo PNDs generally (7, 13, 24). Thus, the pathogenic mechanism of anti-Yo PNM is likely similar to anti-Yo PCD. Indeed, consistent with tumors from patients with anti-Yo PCD, CDR2L was highly expressed in our patient's HER2+ breast carcinoma tissue. The degree of dysfunction in different tissues in individual patients may reflect variation in MHC peptide presentation in different anatomic regions (30).

Standard clinical autoantibody testing fails to identify a diagnostic antibody in up to one third of patients with PNDs, some of whom may harbor undetected classified autoantibodies (25). Few laboratories use multiple modalities upon initial screening for paraneoplastic autoantibodies, and single modality screening is known to risk false positive and negatives for some antibodies including anti-Yo (31). We previously showed that PhIP-Seq significantly enriched CDR2L and/or CDR2 peptides in all 36 screened cases of clinically diagnosed anti-Yo PCD (51 of 53 biospecimens) with no difference in sensitivity between serum and CSF (7). This study extends those data to show that PhIP-Seq may identify paraneoplastic cases for which tissue-based assays are insufficiently sensitive. Together with prior literature, our data suggests a need for complementary autoantibody testing to account for differential target epitope availability among diagnostic tests.

## Patient perspective

Our patient noted that having undiagnosed progressive symptoms increased her general sense of anxiety. She was relieved when immunosuppression provided some relief and was grateful for the opportunity to participate in research that helped clarify the likely cause of her disease.

## Data Availability Statement

The original contributions presented in the study are included in the article/Supplementary Material, further inquiries can be directed to the corresponding author.

## Ethics Statement

The studies involving human participants were reviewed and approved by UCSF Human Research Protection Program. The patients/participants provided their written informed consent to participate in this study. The animal study was reviewed and approved by UCSF Office of Research Institutional Animal Care and Use Program. Written informed consent was obtained from the individual(s) for the publication of any potentially identifiable images or data included in this article.

## Author Contributions

CB, TN, and BA performed the laboratory experiments. NP and MK evaluated the patient, and KZ and JA enrolled the patient. MP interpreted the tumor pathology. SP and MW supervised the study and data analysis. CB, NP, SP, and MW wrote the first draft of the paper. All authors provided critical editing of the text.

## Funding

Confocal microscopy with the CSU-W1 spinning disk was supported by the S10 Shared Instrumentation grant (1S10OD017993-01A1). Tumor histology was supported in part by the University of California, San Francisco Histology & Biomarker Core, a subgroup of the Helen Diller Family Comprehensive Cancer Center Biorepository and Tissue Biomarker Technology Core (BTBMT). The BTBMT was supported by the National Cancer Institute of the National Institutes of Health under Award Number P30CA082103. This work was further supported by: NIMH R01MH122471 (SP, MW, and KZ), NINDS 2R25NS070680-11 (NP), NINDS K08NS096117 (MW), Brain Research Foundation (SP). CB was supported by a Hanna H. Gray Fellowship, Howard Hughes Medical Institute, President's Postdoctoral Fellowship Program, the University of California, the John A. Watson Scholar Program, the University of California, San Francisco, and the Latinx Center of Excellence Grant no. D34HP3178.

## Conflict of Interest

MW receives unrelated research support from Roche/Genentech. The remaining authors declare that the research was conducted in the absence of any commercial or financial relationships that could be construed as a potential conflict of interest.

## Publisher's Note

All claims expressed in this article are solely those of the authors and do not necessarily represent those of their affiliated organizations, or those of the publisher, the editors and the reviewers. Any product that may be evaluated in this article, or claim that may be made by its manufacturer, is not guaranteed or endorsed by the publisher.

## References

[1] Graus F Titulaer MJ Balu R Benseler S Bien CG Cellucci T. A clinical approach to diagnosis of autoimmune encephalitis. Lancet Neurol. (2016) 15:391–404. 10.1016/S1474-4422(15)00401-9 26906964 PMC5066574

[2] Larman HB Zhao Z Laserson U Li MZ Ciccia A Gakidis MAM. Autoantigen discovery with a synthetic human peptidome. Nat Biotechnol. (2011) 29:535. 10.1038/nbt.1856 21602805 PMC4169279

[3] Larman HB Salajegheh M Nazareno R Lam T Sauld J Steen H. Cytosolic 5′-nucleotidase 1A autoimmunity in sporadic inclusion body myositis. Ann Neurol. (2013) 73:408–18. 10.1002/ana.23840 23596012

[4] Larman HB Laserson U Querol L Verhaeghen K Solimini NL Xu GJ. PhIP-Seq characterization of autoantibodies from patients with multiple sclerosis, type 1 diabetes and rheumatoid arthritis. J Autoimmun. (2013) 43:1–9. 10.1016/j.jaut.2013.01.013 23497938 PMC3677742

[5] Xu GJ Shah AA Li MZ Xu Q Rosen A Casciola-Rosen L. Systematic autoantigen analysis identifies a distinct subtype of scleroderma with coincident cancer. Proc National Acad Sci USA. (2016) 113:e7526–34. 10.1073/pnas.1615990113 27821747 PMC5127349

[6] Mandel-Brehm C Dubey D Kryzer TJ O'Donovan BD Tran B Vazquez SE. Kelch-like Protein 11 Antibodies in Seminoma-Associated Paraneoplastic Encephalitis. New Engl J Med. (2019) 381:47–54. 10.1056/NEJMoa1816721 31269365 PMC6800027

[7] O'Donovan B Mandel-Brehm C Vazquez SE Liu J Parent AV Anderson MS. High resolution epitope mapping of anti-Hu and anti-Yo autoimmunity by programmable phage display. Brain Commun. (2020) 2:fcaa059. 10.1093/braincomms/fcaa059 32954318 PMC7425417

[8] Tan PH Ellis I Allison K Brogi E Fox SB Lakhani S. WHO classification of tumours editorial board. The 2019 World Health Organization classification of tumours of the breast. Histopathology. (2020) 77:181–185. 10.1111/his.14091 32056259

[9] Stone JB DeAngelis LM. Cancer-treatment-induced neurotoxicity—focus on newer treatments. Nat Rev Clin Oncol. (2016) 13:92–105. 10.1038/nrclinonc.2015.152 26391778 PMC4979320

[10] Shah S Campo RVD Kumar N McKeon A Flanagan EP Klein C. Paraneoplastic myeloneuropathies: clinical, oncologic, and serologic accompaniments. Neurology. (2020) 96:e632–9. 10.1212/WNL.0000000000011218 33208548 PMC7905784

[11] Pestronk A Schmidt RE Choksi RM Sommerville RB Al-Lozi MT. Clinical and laboratory features of neuropathies with serum IgM binding to TS-HDS. Muscle Nerve. (2012) 45:866–72. 10.1002/mus.23256 22581541

[12] Samara V Sampson J Muppidi S. FGFR3 Antibodies in Neuropathy. J Clin Neuromuscul Dis. (2018) 20:35–40. 10.1097/CND.0000000000000221 30124558

[13] Kråkenes T Herdlevær I Raspotnig M Haugen M Schubert M Vedeler CA. CDR2L is the major yo antibody target in paraneoplastic cerebellar degeneration. Ann Neurol. (2019) 86:316–21. 10.1002/ana.25511 31148214

[14] Uhlén M Fagerberg L Hallström BM Lindskog C Oksvold P Mardinoglu A. Tissue-based map of the human proteome. Science. (2015) 347:1260419. 10.1126/science.1260419 25613900

[15] Loibl S Gianni L. HER2-positive breast cancer. Lancet. (2017) 389:2415–29. 10.1016/S0140-6736(16)32417-5 27939064

[16] Rojas-Marcos I Picard G Chinchón D Gelpi E Psimaras D Giometto B. Human epidermal growth factor receptor 2 overexpression in breast cancer of patients with anti-Yo–associated paraneoplastic cerebellar degeneration. Neuro Oncol. (2012) 14:506–10. 10.1093/neuonc/nos006 22351748 PMC3309857

[17] Small M Treilleux I Couillault C Pissaloux D Picard G Paindavoine S. Genetic alterations and tumor immune attack in Yo paraneoplastic cerebellar degeneration. Acta Neuropathol. (2018) 135:569–79. 10.1007/s00401-017-1802-y 29299667

[18] Venkatraman A Opal P. Paraneoplastic cerebellar degeneration with anti-Yo antibodies – a review. Ann Clin Transl Neur. (2016) 3:655–63. 10.1002/acn3.328 27606347 PMC4999597

[19] Flanagan EP McKeon A Lennon VA Kearns J Weinshenker BG Krecke KN. Paraneoplastic isolated myelopathy. Neurology. (2011) 76:2089–95. 10.1212/WNL.0b013e31821f468f 21670438

[20] Höftberger R Rosenfeld MR Dalmau J. Update on neurological paraneoplastic syndromes. Curr Opin Oncol. (2015) 27:489–95. 10.1097/CCO.0000000000000222 26335665 PMC4640358

[21] Herdlevær I Haugen M Mazengia K Totland C Vedeler C. Paraneoplastic cerebellar degeneration: the importance of including CDR2L as a diagnostic marker. Neurology. (2021) 8:e963. 10.1212/NXI.0000000000000963 33531379 PMC8057066

[22] Ruiz-García R Martínez-Hernández E Saiz A Dalmau J Graus F. The diagnostic value of onconeural antibodies depends on how they are tested. Front Immunol. (2020) 11:1482. 10.3389/fimmu.2020.01482 32760403 PMC7372120

[23] Schwenkenbecher P Chacko L Pul R Sühs K-W Wegner F Wurster U. Paraneoplastic cerebellar syndromes associated with antibodies against Purkinje cells. Int J Neurosci. (2017) 128:1–19. 10.1080/00207454.2017.1412967 29199513

[24] Eichler TW Totland C Haugen M Qvale TH Mazengia K Storstein A. CDR2L antibodies: a new player in paraneoplastic cerebellar degeneration. PLoS ONE. (2013) 8:e66002. 10.1371/journal.pone.0066002 23823982 PMC3688866

[25] Storstein A Monstad SE Haugen M Mazengia K Veltman D Lohndal E. Onconeural antibodies: improved detection and clinical correlations. J Neuroimmunol. (2011) 232:166–70. 10.1016/j.jneuroim.2010.10.009 21093932

[26] McKeon A Tracy JA Pittock SJ Parisi JE Klein CJ Lennon VA. Purkinje cell cytoplasmic autoantibody type 1 accompaniments: the cerebellum and beyond. Arch Neurol-Chicago. (2011) 68:1282–9. 10.1001/archneurol.2011.128 21670387

[27] Othman T Hendizadeh M-S Vankina R Park S Kim P. Combined cerebellar and spinal cord deficits caused by an underlying gynecologic malignancy. Case Rep Oncol Med. (2020) 2020:1–3. 10.1155/2020/9021843 31970005 PMC6973182

[28] Plantone D Caliandro P Iorio R Frisullo G Nociti V Patanella AK. Brainstem and spinal cord involvement in a paraneoplastic syndrome associated with anti-Yo antibody and breast cancer. J Neurol. (2011) 258:921–2. 10.1007/s00415-010-5831-x 21082321

[29] Verschuuren J Chuang L Rosenblum MK Lieberman F Pryor A Posner JB. Inflammatory infiltrates and complete absence of Purkinje cells in anti-Yo-associated paraneoplastic cerebellar degeneration. Acta Neuropathol. (1996) 91:519–25. 10.1007/s004010050460 8740233

[30] Marcu A Bichmann L Kuchenbecker L Kowalewski DJ Freudenmann LK Backert L. HLA Ligand Atlas: a benign reference of HLA-presented peptides to improve T-cell-based cancer immunotherapy. J Immunother Cancer. (2021) 9:e002071. 10.1136/jitc-2020-002071 33858848 PMC8054196

[31] Graus F Vogrig A Muñiz-Castrillo S Antoine J-CG Desestret V Dubey D. Updated diagnostic criteria for paraneoplastic neurologic syndromes. Neurology. (2021) 8:e1014. 10.1212/NXI.0000000000001014 34006622 PMC8237398

