# Corrigendum: Case Report: A False Negative Case of Anti-Yo Paraneoplastic Myelopathy

**DOI:** 10.3389/fneur.2022.888513

**Published:** 2022-03-23

**Authors:** Christopher M. Bartley, Neelroop N. Parikshak, Thomas T. Ngo, Jessa A. Alexander, Kelsey C. Zorn, Bonny D. Alvarenga, Min K. Kang, Massimo Pedriali, Samuel J. Pleasure, Michael R. Wilson

**Affiliations:** ^1^Weill Institute for Neurosciences, University of California, San Francisco, San Francisco, CA, United States; ^2^Department of Psychiatry and Behavioral Sciences, University of California, San Francisco, San Francisco, CA, United States; ^3^Department of Neurology, University of California, San Francisco, San Francisco, CA, United States; ^4^Department of Biochemistry and Biophysics, University of California, San Francisco, San Francisco, CA, United States; ^5^Operative Unit of Surgical Pathology, Azienda Ospedaliera-Universitaria, Ferrara, Italy

**Keywords:** paraneoplastic neurologic disease, myelopathy, anti-Yo antibodies, phage display, breast cancer, CDR2L

An author name was incorrectly spelled as Bonny A. Alvarenga. The correct spelling is Bonny D. Alvarenga.

The authors apologize for this error and state that this does not change the scientific conclusions of the article in any way. The original article has been updated.

## Publisher's Note

All claims expressed in this article are solely those of the authors and do not necessarily represent those of their affiliated organizations, or those of the publisher, the editors and the reviewers. Any product that may be evaluated in this article, or claim that may be made by its manufacturer, is not guaranteed or endorsed by the publisher.

