# Supplementary material for: Case Report: A False Negative Case of Anti-Yo Paraneoplastic Myelopathy
Source: Front Neurol. 2021 Oct 22;12:728700. doi: 10.3389/fneur.2021.728700 (PMC8570369; doi:10.3389/fneur.2021.728700)
Supplement: Supplementary file 1 [file Data_Sheet_1.docx]

**Supplemental Data**

**Supplemental Table 1**

| **Months from breast cancer diagnosis** | **Study** | **Details** |
| --- | --- | --- |
| **Imaging studies and biopsies** | | |
| 0 | Left breast US guided biopsy | Found to have breast cancer |
| 1 | PET scan | Revealed no evidence of systemic metastases |
| 1 | Left sentinel node biopsy | Negative biopsy |
| 14 | CT head | Done for reported instability with walking and fall at home. Normal study |
| 17 | MR brain +/- contrast | Normal study |
| 19 | MR lumbar spine +/- contrast | Normal study |
| 27 | MR C & T spine without contrast | Longitudinally extensive T2 FLAIR signal in T spine spanning T5-T10 |
| 30 | PET/CT whole body vertex to toe | No evidence of hypermetabolic lesions concerning for recurrent or metastatic disease |
| 33 | MR C & T spine +/- contrast | Improvement in the long segment T2 hyperintensity predominantly involving the dorsal thoracic cord, spanning T7-T10 but now conspicuous only at the T7-T8 level. No abnormal contrast enhancement |
| **Electrodiagnostic studies** | | |
| 15 | Tibial somatosensory evoked potentials (SSEPs) | Reported abnormal study from outside hospital |
| 15 | EMG/NCS | Normal study |
| 17 | Repeat EMG/NCS | Normal study, no evidence of neuropathy |
| 20 | Median and tibial SSEPs | Normal study for median nerve, abnormal for tibial |
| 24 | Repeat median and tibial SSEPs | Normal study for median nerve, abnormal for tibial |
| 24 | Repeat EMG/NCS | Normal study, no evidence of neuropathy |
| **Serum studies** | | |
| 16 | HIV Ag/Ab negative, HgbA1c | Within normal limits |
| 17 | Serum protein electrophoresis with immunofluorescence and kappa/lambda free light chains, plasma copper, TSH, and vitamin B12 | Within normal limits |
| 20 | Mayo autoimmune encephalitis panel | Within normal limits, no reflex testing indicated |
| 22 | Washington University neuromuscular antibody panel | Following abnormal values: IgM vs TS-HDS 48000 (normal < 10000), IgG vs FGFR3 3800 (normal < 3000), IgG vs beta-tubulin 3000 (normal < 2500) |
| 26 | Vitamin E | Within normal limits |
| 34 | NMO/MOG antibodies, syphilis reactivity, copper, folate, B12, methylmalonic acid, tocopherols, angiotensin-converting enzyme, tissue transglutaminase, rheumatoid factor, cytomegalovirus IgM, dsDNA, SSA/SSB, HTLV I/II antibodies | All studies within normal limits with exception ofdsDNA, SSA/SSB, and HTLV I/II antibodies, which were positive as labs were drawn within a week of recent IVIG administration |
| 37 | Redraw of dsDNA, SSA/SSB, and HTLV-I/II negative, this time about one month after IVIG administration | Within normal limits |
| **Cerebrospinal fluid studies** | | |
| 18 | Cell count, IgG index, oligoclonal bands, cytology, flow cytometry | Noninflammatory CSF (WBC 2, RBC 0, glucose 57, protein 29, IgG index within normal limits, no oligoclonal bands). Cytology and flow cytometry negative. |
| 30 | Repeat cell count, igG index, oligoclonal bands, cytology, flow cytometry | Within normal limits |
| 36 | Repeat CSF studies as prior, now including CSF VDRL, Mayo autoimmune encephalitis panel | Within normal limits |

**Supplemental Table 2.**

| **Rank** | **Peptide ID** | **Replicate 1** | **Replicate 2** |
| --- | --- | --- | --- |
| 1 | **CDR2L_292152** | 1464.285 | 1012.506 |
| 2 | ADAMTSL2_638488 | 659.0086 | 112.4453 |
| 3 | TACC2_571590 | 708.9254 | 22.63139 |
| 4 | SF3A1_224642 | 103.4054 | 430.9216 |
| 5 | **CDR2L_292161** | 28.03912 | 438.8212 |
| 6 | CCDC93_117335 | 271.0151 | 149.3814 |
| 7 | ZNF467_613542 | 190.5589 | 160.1278 |
| 8 | SCN11A_132220 | 263.8713 | 52.45074 |
| 9 | LOC101928841_690498 | 82.33143 | 190.3029 |
| 10 | CCDC51_143976 | 85.27821 | 160.6259 |
| 11 | ANKRD24_3299 | 188.9515 | 56.64964 |
| 12 | HERC2_92437 | 8.03669 | 227.0256 |
| 13 | ZRSR2_53276 | 197.1668 | 15.15876 |
| 14 | EPB41_379703 | 148.8574 | 57.29015 |
| 15 | SUN1_538724 | 170.3778 | 23.12957 |
| 16 | ARMC5_709181 | 185.9154 | 6.476278 |
| 17 | TMEM191B_41962 | 12.59081 | 178.4179 |
| 18 | KCNN3_287981 | 143.0531 | 43.05658 |
| 19 | TMEM263_475642 | 138.8561 | 37.29198 |
| 20 | RNF157_718401 | 47.68436 | 119.2774 |
| 21 | CD3EAP_527253 | 32.77184 | 128.1734 |
| 22 | ZBTB21_38724 | 124.3008 | 22.41789 |
| 23 | **CDR2_373776** | 27.77123 | 117.2847 |
| 24 | KIF26A_278687 | 130.8195 | 12.81022 |
| 25 | MLLT4_606155 | 14.55534 | 120.8431 |
| 26 | TLN1_417263 | 70.27639 | 63.8376 |
| 27 | PLEC_625830 | 25.9853 | 107.3212 |
| 28 | ATXN3_576289 | 77.7773 | 51.16972 |
| 29 | LOC101928722_371120 | 57.86417 | 61.34672 |
| 30 | EPS8L1_263542 | 37.41526 | 76.07848 |
| 31 | SART3_555360 | 42.50516 | 68.10767 |
| 32 | TMEM102_575717 | 57.59628 | 50.95621 |
| 33 | NEK1_172328 | 20.53821 | 87.25184 |
| 34 | BAHCC1_465605 | 54.73879 | 51.81023 |
| 35 | MAMSTR_17148 | 61.70392 | 43.41242 |
| 36 | SPARC_583054 | 8.215283 | 95.36498 |
| 37 | COL21A1_601920 | 77.50941 | 24.76643 |
| 38 | CAMK2B_611516 | 82.59932 | 16.79562 |
| 39 | TDRP_619245 | 52.86356 | 43.76825 |
| 40 | EHMT1_637164 | 19.19876 | 72.23541 |
| 41 | ARHGAP27_71523 | 25.27093 | 65.75913 |
| 42 | ABLIM2_496737 | 77.33082 | 11.03102 |
| 43 | RNF151_704343 | 49.47029 | 37.50548 |
| 44 | WISP1_205531 | 33.6648 | 49.60402 |
| 45 | CCDC177_308946 | 66.97242 | 13.5219 |
| 46 | OSCP1_377170 | 8.215283 | 71.45257 |
| 47 | VWA5B2_158526 | 20.6275 | 56.93431 |
| 48 | ZNF618_631338 | 70.18709 | 6.974454 |
| 49 | MDC1_68205 | 19.91313 | 56.36497 |
| 50 | UNC80_111964 | 25.18163 | 50.67154 |
| 51 | PERM1_468094 | 39.0226 | 35.44161 |
| 52 | LRRFIP1_129573 | 39.0226 | 34.72993 |
| 53 | SUN1_538858 | 57.32839 | 13.94891 |
| 54 | KIF13A_196932 | 48.22014 | 18.00548 |
| 55 | SPOCD1_460451 | 12.76941 | 52.66424 |
| 56 | B4GALNT4_499668 | 44.0232 | 21.2792 |
| 57 | TANC1_127969 | 11.07277 | 48.18066 |
| 58 | FBF1_226776 | 34.37917 | 24.69526 |
| 59 | ARL6IP4_440311 | 17.85931 | 40.99271 |
| 60 | KMT2D_674501 | 29.19997 | 28.75183 |
| 61 | SOX17_250523 | 46.88069 | 10.95986 |
| 62 | CAB39_116981 | 43.13024 | 13.94891 |
| 63 | PRPF40B_669998 | 32.95043 | 22.63139 |
| 64 | LOC105376902_27051 | 34.37917 | 20.92336 |
| 65 | UBOX5_445765 | 49.2917 | 5.05292 |
| 66 | ASGR1_265785 | 20.35962 | 32.73723 |
| 67 | MUC5AC_570082 | 40.80853 | 10.95986 |
| 68 | PYGL_515110 | 18.57368 | 33.16424 |
| 69 | FIP1L1_165970 | 38.39752 | 12.38321 |
| 70 | CD320_521711 | 8.393876 | 40.99271 |
| 71 | EIF2B4_214217 | 11.51926 | 36.7938 |
| 72 | FHAD1_338599 | 23.3064 | 22.91606 |
| 73 | RFTN1_135472 | 24.82444 | 20.85219 |
| 74 | PPTC7_592761 | 22.32414 | 23.20073 |
| 75 | CLIC6_39520 | 26.96756 | 16.65329 |
| 76 | SRRM2_578712 | 7.947394 | 35.58395 |
| 77 | CDK13_534820 | 30.62872 | 10.60402 |
| 78 | FRMD8_286484 | 5.000607 | 36.08212 |
| 79 | HOXB4_492713 | 33.30762 | 6.191607 |
| 80 | CCDC88B_656716 | 29.82505 | 9.10949 |
| 81 | SNCG_436752 | 31.2538 | 7.472629 |
| 82 | PRSS56_349002 | 26.43178 | 11.38686 |
| 83 | NUDT9_264849 | 13.57308 | 24.19708 |
| 84 | AXIN1_706469 | 16.34127 | 19.71351 |
| 85 | DCLRE1B_554643 | 24.37796 | 11.38686 |
| 86 | LOC105370548_372920 | 15.26971 | 19.99818 |
| 87 | HAS1_18079 | 23.3064 | 11.5292 |
| 88 | ALDH7A1_387936 | 15.26971 | 18.00548 |
| 89 | RASL11A_406935 | 24.11007 | 7.7573 |
| 90 | EGLN1_87324 | 16.25197 | 12.66789 |
| 91 | TSGA10IP_292003 | 16.25197 | 11.38686 |
| 92 | CRNKL1_460951 | 15.5376 | 10.03467 |
| 93 | PLEKHA6_687529 | 8.57247 | 16.79562 |
| 94 | MKL2_705800 | 19.82384 | 5.05292 |
| 95 | XYLT1_533006 | 6.250759 | 18.21898 |
| 96 | GPRASP2_260851 | 10.4477 | 14.02008 |
| 97 | PHC1_664973 | 19.10946 | 5.337592 |
| 98 | RSRC2_673514 | 16.51986 | 6.476278 |
| 99 | RSPH3_524294 | 12.76941 | 10.03467 |
| 100 | SLC45A2_248041 | 15.18041 | 5.124088 |
| 101 | TCP10L2_286040 | 11.16207 | 8.611315 |
| 102 | DTD1_236551 | 6.429352 | 10.31934 |
| 103 | LAMA5_33434 | 8.125987 | 8.11314 |
| 104 | BCORL1_62057 | 10.17981 | 5.835767 |
| 105 | NCOA3_488600 | 10.80488 | 5.195256 |
| 106 | LILRB2_12684 | 8.929656 | 5.978103 |
| 107 | ASXL1_31760 | 8.751063 | 6.049271 |
| 108 | PM20D2_210546 | 5.71498 | 6.974454 |
| 109 | PREX1_532686 | 5.1792 | 5.266424 |

**Supplemental Table 2. PhIP-Seq results for patient CSF.** Patient CSF was screened by PhIP-Seq in technical replicate. Show are reads per hundred thousand (rpK) for peptides with a minimum rpK in each replicate that were enriched at least 10-fold over reference CSF samples. Peptides are ranked in descending order by rpK. Peptide IDs are formatted as GENE_index, whereby index represents a unique peptide within the PhIP-Seq library.

**Supplementary Figure 1**


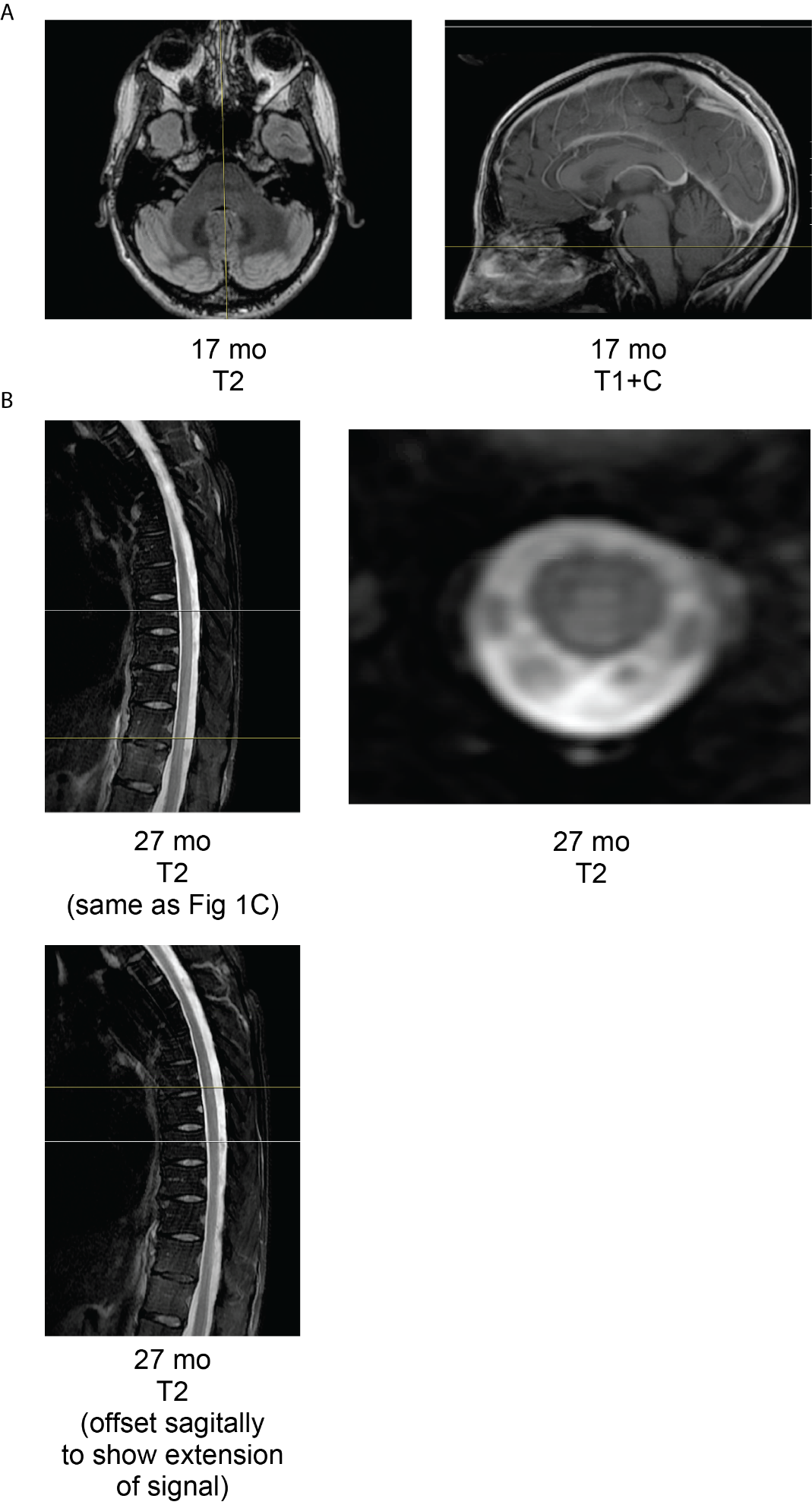


**Supplementary Figure 1. Additional neuroimaging. A.** MRI brain demonstrating a lack of cerebellar atrophy or contrast enhancement in brain. **B.** Further views of thoracic spine imaging shown in Figure 1, demonstrating extension of lesion from T5-T10 (primary figure shows T6-T10 due to remainder of signal being out-of-plane).


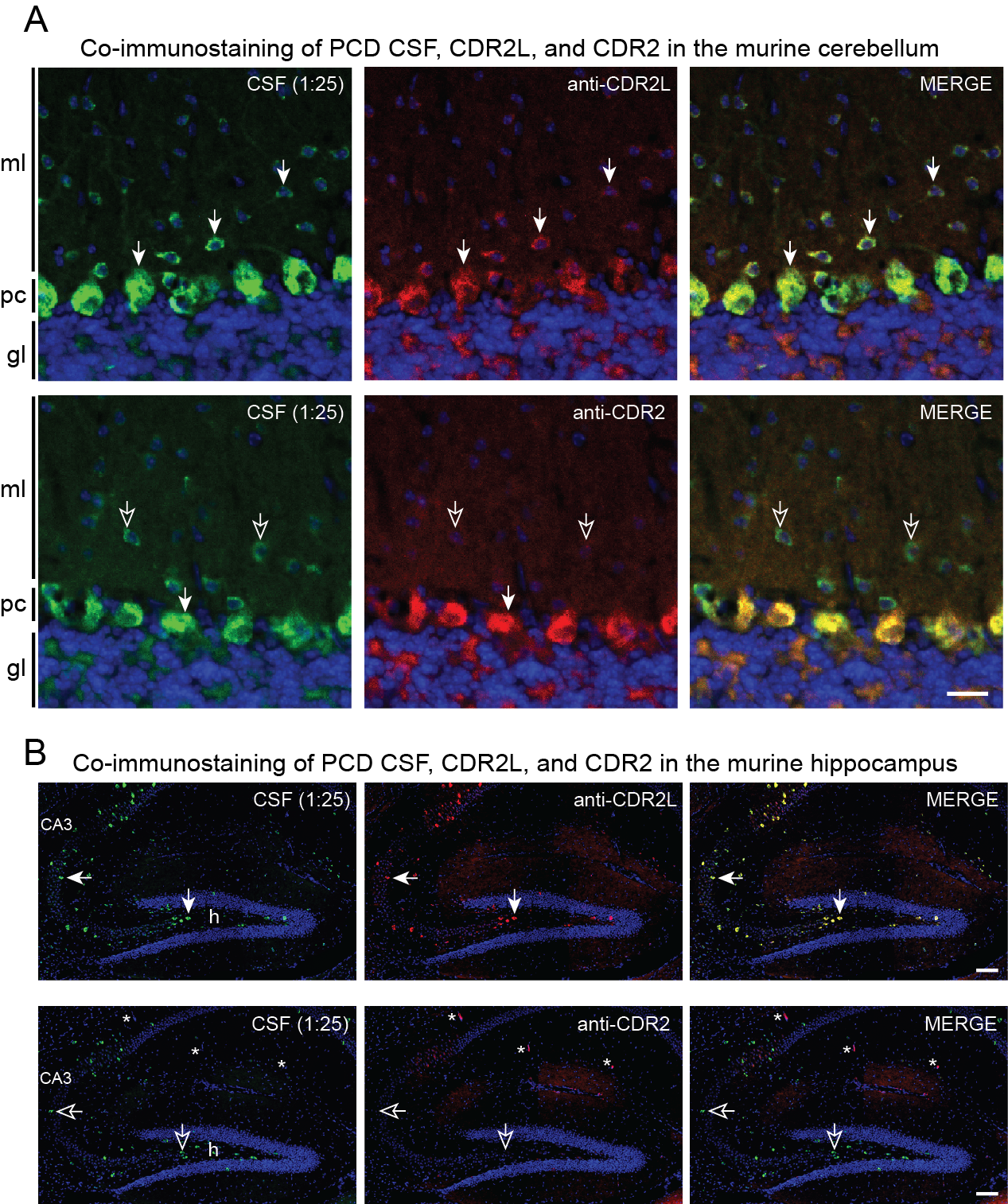


**Supplementary Figure 2. Co-immunostaining of PCD CSF and CDR2L and CDR2 in the murine brain. A.** Mouse brain cerebellum coimmunostained with PCD CSF (1:25) and anti-CDR2L or anti-CDR2. In the top row, the arrows point to PCs and molecular layer neurons that are immunostained by both CDR2L and PCD CSF. In the lower row, PCs are immunostained by CSF and CDR2 (filled arrows) but molecular layers neurons are only immunostained by PCD CSF (unfilled arrows). On the left, ml = molecular layer, pc = Purkinje cell layer, gl = granule cell layer. **B.** Coimmunostaining of the mouse hippocampus. In the top row, cells in CA3 and the hilus were coimmunostained by PCD CSF and anti-CDR2L (filled arrows). In the lower CA3 and hilar cells were immunostained by PCD CSF but not anti-CDR2 (unfilled arrows). Asterisks indicate CDR2+ blood vessels.
